# Influenza vaccination, inverse care and homelessness: cross-sectional survey of eligibility and uptake during the 2011/12 season in London

**DOI:** 10.1186/1471-2458-14-44

**Published:** 2014-01-16

**Authors:** Alistair Story, Robert W Aldridge, Tat Gray, Stan Burridge, Andrew C Hayward

**Affiliations:** 1 Find & Treat, University College Hospitals NHS Foundation Trust, 2nd Floor - Hospital of Tropical Diseases, Mortimer Market Centre, 5th Floor East, 250 Euston Rd, London NW1 2PG, UK; 2 Research Department of Infection & Population Health, University College London, First Floor, Royal Free Hospital, Rowland Hill St, London NW3 2PF, UK

## Abstract

**Background:**

Influenza vaccination eligibility and uptake among homeless adults has not been previously assessed in the UK. This cross-sectional survey aimed to measure the proportion of homeless people visited by an NHS outreach service (Find and Treat) who were eligible for and had received vaccination during 2011/12.

**Methods:**

A cross-sectional survey was carried out in 27 separate homeless hostels, day centres and drug services in London between July and August in 2012. Eligibility for the survey was by virtue of being in attendance at one of 27 venues visited by Find and Treat. No specific exclusion criteria were used.

**Results:**

455 clients took part in the survey out of 592 approached (76.9%). A total of 190 homeless people (41.8%; 95% CI: 34.5,50.5) were eligible for influenza vaccination. In those aged 16–64, eligibility due to clinical risk factors was 38.9% (95% CI: 31.5,48.2). Uptake of vaccination in homeless 16–64 year olds with a clinical risk factor during the 2011/12 influenza season was 23.7% (95% CI: 19.8,28.3) compared to national levels of 53.2% (excluding pregnant women). In those aged over 65, uptake was 42.9% (95% CI: 16.7,100.0) compared with 74.0% nationally.

**Conclusions:**

This study demonstrates that the homeless population have high levels of chronic health problems predisposing them to severe complications of influenza, but vaccine uptake levels that are less than half those seen among eligible GP patient groups in England. It provides a clear example of the health inequalities and inverse care law that impact this population. The results of this study provide strong justification for intensifying efforts to ensure homeless people have access to influenza vaccination.

## Background

Homelessness is the epitome of exclusion and health inequality and is an independent risk factor for premature mortality [1]. Around 100,000 people in the UK cycle through the hostel system annually, many having been previously rough sleeping [2]. Health care usage among homeless people in England is characterised by unplanned and emergency care. Compared with patients who are not homeless they attend accident and emergency departments 5 times as often, are admitted to hospital 3.2 times as often, and stay three times as long, resulting in unscheduled secondary care costs that are 8 times higher [3]. Mortality from respiratory infections is 7 times greater among homeless people [4]. This is compounded by a high prevalence of chronic respiratory diseases [5] and high rates of smoking tobacco [6] and illicit drugs. Homeless adults are at greater risk of vaccine preventable respiratory infections due to a high prevalence of underlying medical conditions and increased transmission risk associated with congregate living in confined shared air spaces [7].

In England, influenza vaccination is recommended for all those aged 65 years and over and all those aged six months to under 65 years falling into one or more of the following clinical risk categories (Table 1).

**Table 1:** **Eligibility Criteria for Influenza Vaccination **[8]

**Risk category**	Examples*
Chronic respiratory disease	Asthma that requires continuous or repeated use of inhaled or systemic steroids or with previous exacerbations requiring hospital admission. Chronic obstructive pulmonary disease (COPD) including chronic bronchitis and emphysema; bronchiectasis, cystic fibrosis, interstitial lung fibrosis, pneumoconiosis and bronchopulmonary dysplasia (BPD).
Chronic heart disease	Congenital heart disease, hypertension with cardiac complications, chronic heart failure, individuals requiring regular medication and/or follow-up for ischaemic heart disease.
Chronic renal disease	Chronic kidney disease at stage 3, 4 or 5, chronic kidney failure, nephrotic syndrome, kidney transplantation.
Chronic liver disease	Cirrhosis, biliary atresia, chronic hepatitis.
Chronic neurological disease	Stroke, transient ischaemic attack (TIA). Conditions in which respiratory function may be compromised due to neurological disease.
Diabetes	Type 1 diabetes, type 2 diabetes requiring insulin or oral hypoglycaemic drugs, diet controlled diabetes.
Immunosuppression	Immunosuppression due to disease or treatment.
Pregnant women	Pregnant women at any stage of pregnancy.

All aged 65 years and over. *Examples are relevant to an adult population and are not exhaustive - decisions should be based on clinical judgement.

During the 2010/11 influenza season, patients with chronic disease in these risk groups had a 10-fold greater risk of mortality due to influenza compared with those who were not in an at-risk group [9]. Among GP patient groups in England, cumulative influenza vaccine uptake from 1 September 2011 to 31 January 2012 was 74.0% for those aged 65 years and over and 51.6% for those aged six months to under 65 years in one or more clinical risk groups (excluding pregnant women without other risk factors). Uptake by individual risk group ranged from 43.3% among patients with chronic liver disease to 68.5% among those with diabetes [10].

Influenza vaccination eligibility and uptake among homeless adults has not been previously assessed in the UK. This cross-sectional survey aimed to measure the proportion of homeless people (attending venues visited by Find and Treat) who were eligible for influenza vaccination due to clinical risk or age, the proportion vaccinated in the 2011/12 influenza season, and where these individuals accessed vaccination services.

## Methods

A cross-sectional survey was carried out during July and August in 2012 at 27 separate venues including homeless hostels, day centres and drug services used by homeless people. Find and Treat is a service that performs active case finding for TB among the homeless population in London, and carries out case management activities for individuals suspected or diagnosed with TB. This survey was performed alongside the clinical work of Find and Treat and therefore convenience sampling was used for choosing venues with no specific sampling framework. Clients were invited to take part in the survey by either one of two members of the Find and Treat staff (both with several years of working with this client group) or a peer health advocate with personal experience of homelessness who works alongside this team.

Training of the interviewers involved them piloting and refining the questionnaire among peer educators who work for the service. Feedback to the interviewers from peers during the pilot process was used to develop a standardised approach to asking questions. The pilot process and data collection was overseen by the clinical lead for the service. Interviewers sat with clients and helped them fill out a questionnaire of survey responses. A copy of the questionnaire used is included in the Additional file 1. Clients were eligible to take part by virtue of being in attendance at one of these venues, and no specific exclusion criteria were used.

Clients were asked to self-report basic demographic information including age, sex, whether they were born in the UK and if they were currently registered with a GP practice. They were also asked to self-report clinical risk factors that would make them eligible for influenza vaccination, willingness to be vaccinated, whether they had received vaccination during the previous 12 months and if so where this was performed. All variables were recorded as either binary or categorical variables. Responses were collected by the three interviewers and manually entered into a database.

The primary outcomes for this study were the proportion of homeless people eligible for influenza vaccination during the 2011/12 influenza season and uptake among those who were eligible. Secondary outcomes were the location in which vaccination had been administered during the 2011/12 influenza season, and GP registration levels. The study was powered to measure what proportion of homeless people were eligible for influenza vaccination and to examine uptake among those eligible in order to inform policy decisions for this group. Sample sizes were increased to take account of clustering by venue assuming an average of 10 recruits per site, an intracluster correlation coefficient (ICC) of 0.05 (based on upper range of ICCs for chronic disease occurrence and uptake of interventions in nursing homes and general practices) giving a design effect of 1.45 [11]. An initial sample size of 200 was chosen to allow an overall eligibility of 50% to be measured with 95% confidence intervals of 42-58%.

Estimates of baseline characteristics, clinical risk factor prevalence and vaccine uptake were adjusted by using STATA cluster commands to take into account potential clustering at the 27 sampling venues which clients were recruited from. The percentage of homeless clients meeting age and clinical eligibility criteria for influenza vaccination, and the percentage that received vaccination during the 2011/12 season, were compared to national data for the same season using figures published by the Department of Health [10]. All data were analysed in STATA version 12. This anonymous survey was carried out by an NHS organisation for the purposes of improving service delivery to its client group and therefore an ethics committee was not approached for approval. The purpose of the questionnaire was explained to all participants prior to taking part in the survey, answering the survey questions implied consent. No questionnaires were completed at venues providing services to minors.

## Results

455 clients took part in the survey out of 592 approached (76.9%). Individuals were interviewed at 11 day centres, 11 hostels and 5 drug services. The minimum number of clients interviewed at a venue was one, the maximum was 75, and the median was 23. The majority of respondents were men (365/452; 80.8%) between the age of 16–44 (261/455; 57.4%) and born in the UK (277/452; 61.3%). Most questionnaires were completed in day centres (235/455; 51.6%). Levels of missing data were less than 1% across all variables (Table 2).

**Table 2:** Baseline characteristics of homeless respondents

	**Total in survey**	
	**N**	**% (95% CI)**
**All**	455	-
**Age**		
16 to 44	261	57.4% (50.6, 65.1)
45 to 64	173	38.0% (33.5, 43.2)
65+	21	4.6% (2.4, 9.1)
**Sex**		
Male	365	80.8% (70.7, 92.2)
Female	87	19.2% (11.0, 33.6)
**UK born**		
No	175	38.7% (29.2, 51.4)
Yes	277	61.3% (51.3, 73.3)
Survey venue*		
Day centre	235	51.6% (31.4, 84.9)
Homeless hostel	149	32.7% (16.1, 66.4)
Drug service	71	15.6% (6.3, 38.6)
**Registered with a local General Practitioner**		
No	123	27.2% (19.2, 38.5)
Yes**	329	72.8% (63.9, 82.9)

Data were missing for three individuals for the Sex, UK Born and Registered with local General Practitioner variables.

*Day centres provide a range services to homeless people; homeless hostels provide accommodation alongside other variable services; and drug services provide treatment and other rehabilitation services to homeless clients.

**A local General Practitioner was defined as one that was easy to travel to.

**Figure 1:**
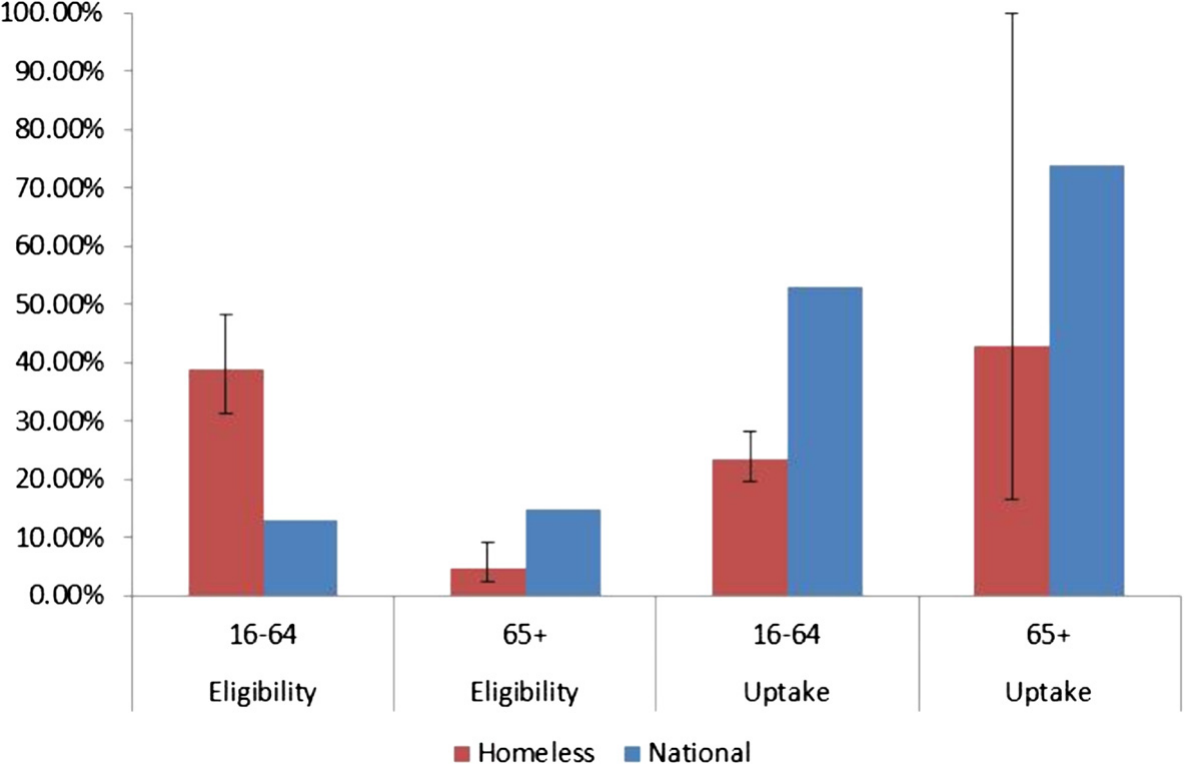
**Proportion of homeless and national population meeting eligibility criteria for influenza vaccination, and uptake levels by age during the 2011/12 influenza season.** Note: Denominator for eligibility in 65+ national reference group is all individuals registered with a GP aged over 16. 4.6% (95% CI :2.3, 9.1 (21/455)) homeless people surveyed were eligible due to age (65+).

A total of 190 (41.8%; 95% CI: 34.5, 50.5) individuals were eligible for influenza vaccination. In those aged 16–64, 169 (38.9%; 95% CI: 31.5, 48.2) were eligible due to clinical risk factors (Table 3 and Figure 1). 21 homeless people (4.6%; 95% CI: 2.3, 9.1) taking part in the survey were eligible as a result of their age (over 65). Almost one-third (52/169; 30.8%) of influenza vaccine eligible homeless people aged 16–64 had two of more clinical risk factors.

**Table 3:** Influenza vaccine risk groups and uptake levels amongst the homeless compared to national rates during the 2011/12 respiratory virus season

	**Homeless**				**National**	
**Risk group**	**n/N**	**% in risk group (95% CI)**	**n/N**	**% of risk group vaccinated (95% CI)**	% in risk group**	**% of risk group vaccinated**
16 to 64 in clinical risk group	169/434	38.9 (31.5, 48.2)	40/169	23.7 (19.8, 28.3)	13.0	53.2
Respiratory disease*	96/434	22.1 (17.7, 27.7)	24/96	25.0 (20.5, 30.5)	5.6	52.3
Heart disease*	41/434	9.5 (6.9, 13.0)	15/41	36.6 (25.3, 52.8)	2.1	55.5
Diabetes*#	17/434	3.9 (2.7, 5.7)	8/17	47.1 (25.4, 87.4)	3.2	68.6
Liver disease*	42/434	9.7 (5.2, 17.9)	10/42	23.8 (16.0, 35.4)	0.6	43.4
Degenerative neurological disease*	33/434	7.6 (5.4, 10.7)	9/33	27.3 (19.4, 38.3)	1.3	50.3
65 + #	21/455	4.6 (2.3, 9.1)	9/21	42.9 (16.7, 100.0)	15.0	74.0
Meets any criteria	190/455	41.8 (34.5, 50.5)	49/190	25.8 (19.5, 34.2)	30.7	65.8

*Clinical risk groups in those aged < 65. Totals add up to more than 169 as individuals can have more than one clinical risk factor.

**For 16 to 64 in clinical risk group and individual clinical risk factors (Respiratory disease, Heart disease, Diabetes, Liver disease, Degenerative neurological disease) denominator is all individuals aged 16–64 and registered with a GP. For 65+ and meets any criteria risk groups, denominator is all individuals registered with a GP aged over 16.

#Estimate may not reliable as sample size <30 or 95% CI half width >10.

Uptake of vaccination in homeless 16–64 year olds with a clinical risk factor was 23.7% (95% CI: 19.8, 28.3) during the 2011/12 influenza season compared with 53.2% nationally (excluding pregnant women). In those aged over 65, uptake was 42.9% (95% CI: 16.7, 100.0) compared with 74.0% nationally. For homeless people with clinical risk factors, uptake was highest among those with diabetes (47.1%; 95% CI: 25.4, 87.4) and lowest in those with chronic liver disease (23.8%; 95% CI: 16.0, 35.4). Influenza vaccine uptake was lower than national levels for all clinical risk groups.

Of the 49 eligible homeless individuals who were vaccinated during the previous influenza season, the majority (38) had accessed vaccination services in General Practice, 3 at a pharmacy, 2 in hostels and drug services respectively, 1 whilst a hospital inpatient, 1 at another venue and 2 were not recorded. Almost three quarters (72.8%; 329/452) of the homeless people interviewed were registered with a general practitioner. Among all homeless individuals surveyed who were eligible for influenza vaccine, 73.2% (139/190) said they would accept if offered, and of those not eligible under current recommendations, 66.0% (175/265) said they would accept vaccination if offered.

## Discussion

This analysis presents the first assessment of influenza vaccine eligibility, access and uptake among homeless people in the UK. Our findings are a clear example of health inequalities and illustrate the inverse care law [12]. Among those aged 16–64, nearly 40% were eligible for influenza vaccination due to clinical risk factors compared with 13% nationally. Only a quarter of these were vaccinated compared to over 50% among eligible adults aged less than 65 nationally. Willingness to accept influenza vaccination if offered was very high among eligible homeless people (75%) suggesting that this group could meet the Chief Medical Officer of England’s target of 75% uptake in clinical risk groups, provided appropriate strategies are in place to reach those eligible and to offer them vaccination [13]. In common with national trends, older individuals were more likely to have been vaccinated than those in younger groups, perhaps due to increased awareness of eligibility for vaccination in this group.

This study has some limitations. Participants and investigators were not blinded to the analysis question, which may result in bias. Measures used were self-reported and potentially subject to recall bias. Comparative general population uptake data is based on GP records of clinical diagnostic codes whereas our data are based on self-reporting of chronic illness. Interviewers were highly experienced in communicating with homeless clients and noted that some respondents had difficulty in reliably differentiating between vaccination during the last influenza season and previous seasons. Chronic health problems are commonly under diagnosed among homeless people [14]. This may result in under estimation of vaccine uptake and clinical risk factors, although this is difficult to establish with the current analyses. Three members of staff were trained to carry out the surveys across venues, however, no formal tests of inter-observer reliability were carried out and we cannot rule out the possibility of bias within the responses received. Our findings may not be applicable beyond the study population and geographic area despite a high response rate and efforts to sample respondents from a wide range of services across London. Because of the greater burden of homelessness in London, services for this population are likely to be more highly developed than elsewhere in the country. Levels of clinical need are likely to be as high elsewhere, however, and influenza vaccine uptake is unlikely to be better, and most probably will be lower.

We note that the indications for vaccination of adults against *Streptococcus pneumonia* are similar to those for influenza. We did not ask about streptococcal vaccination as it was felt this would be poorly recalled, as it is not given annually. We would, however, expect similarly low coverage of streptococcal vaccine among eligible homeless people.

Specific recommendations for influenza vaccination amongst homeless people are made in a number of international influenza vaccination policies [15]. In the USA and Canada, influenza vaccination is recommended for anyone ≥6 months of age without contraindications and specific provision is made to ensure high uptake among homeless people and staff in congregate settings [16,17]. In Australia, influenza vaccination is recommended for homeless people and those providing care to them due to living conditions and prevalence of underlying medical conditions that will predispose to complications and transmission of influenza [18]. Initiatives to outreach snapshot vaccination interventions against influenza, HBV, HAV, Streptococcus pneumoniae, and diphtheria to homeless populations in France have also been reported as effective [19,20].

This is a large and representative study which demonstrates that the homeless population have extremely high levels of chronic health problems that predispose to severe complications of influenza with associated hospitalisation and death. The results of this study provide strong justification for intensifying efforts to ensure homeless people have access to influenza vaccination. Homeless people have high rates of chronic disease, much of which is undiagnosed, high mortality from respiratory infection and often live in congregate settings likely to enhance transmission of influenza. A policy to vaccinate all homeless people living in hostels, accessing daycentres and drug services for influenza, regardless of clinical risk group, is needed. A universal influenza vaccination campaign is likely to result in greater uptake in those with diagnosed and undiagnosed chronic disease and would have the potential benefit of reducing the risk of institutional outbreaks of influenza that occur in congregate settings such as hostels and day centres [21]. Frontline staff working with homeless people could be considered to be covered under existing recommendations to vaccinate health and social care staff, although to our knowledge very few staff are offered vaccination on this basis [8].

Recommendations on how to increase influenza vaccine uptake among clinical risk groups are likely to be challenging to implement for homeless populations [22] and may promote intervention-induced-inequality unless specific provision is made to target this vulnerable population. Homeless people have high levels of multi-morbidity with physical disease and psychological problems often compounded by substance misuse and, by definition, poverty and homelessness. Despite this our work demonstrates this group are no less willing to accept the offer of influenza vaccination than people without these multiple problems. The authors are now working in partnership with Homeless Link, the national umbrella organisation for frontline homelessness services in England, and the Faculty for Homeless and Inclusion Health to launch a national influenza awareness campaign targeted at homeless people and staff in hostels and day centres.

## Conclusion

Compared to the general population, homeless people aged 16–64 are nearly three times more likely to be eligible for influenza vaccination with nearly 40% having clinical risk factors compared with 13% at national level. Despite this only a quarter of eligible homeless adults aged 16–64 were vaccinated compared to over 50% nationally. Primary care services will play a key role in enabling homeless people to access influenza vaccine, but specific targeted snapshot interventions to outreach vaccination are needed to achieve a coverage of 75% or greater. Our findings are a clear example of health inequalities and illustrate the inverse care law: “The availability of good medical care tends to vary inversely with the need for it in the population served. This inverse care law operates more completely where medical care is most exposed to market forces” [12]. This is of particular concern at present given the current reforms to the NHS competition regulations [23].

## Competing interests

All authors have completed the Unified Competing Interest form (http://www.icmje.org/coi_disclosure.pdf - available on request from the corresponding author) and declare: TG and SB had financial support from Knowledge Into Action Registered Charity No for support in completing submitted work; no other financial relationships exist with any organisations that might have an interest in the submitted work in the previous 3 years; no other relationships or activities exist that could have influenced the submitted work. RWA was funded by a Wellcome Trust research training fellowship WT097980MA. AS is funded by UCLH Foundation Trust. AH is funded by Central and North West London NHS Foundation Trust.

## Authors’ contributions

AS and ACH came up with the conception and design of the study, designed data collection tools, and monitored data collection. RWA cleaned the data. AS, RWA and ACH analysed the data and all authors were involved in the interpretation of the data. AS, RWA and ACH drafted the initial paper and all authors were involved in revising it, critically appraising it for important intellectual content, and providing final approval of the version to be published. All authors, had full access to all of the data in the study and can take responsibility for the integrity of the data and the accuracy of the data analysis. ACH is guarantor.

## Pre-publication history

The pre-publication history for this paper can be accessed here:

http://www.biomedcentral.com/1471-2458/14/44/prepub

## Supplementary Material

Additional file 1 TB Reach/Find & Treat Anonymous Health Survey. Click here for file
