# Supplementary material for: Influenza vaccination, inverse care and homelessness: cross-sectional survey of eligibility and uptake during the 2011/12 season in London
Source: BMC Public Health. 2014 Jan 16;14:44. doi: 10.1186/1471-2458-14-44 (PMC3906096; doi:10.1186/1471-2458-14-44)
Supplement: Additional file 1 — TB Reach/Find & Treat Anonymous Health Survey. [file 1471-2458-14-44-S1.pdf]

TB Reach/Find & Treat Anonymous Health Survey

A) Demographics

1 Sex
- ☐ Male
- ☐ Female

2 Age
- ☐ 16-24
- ☐ 25-34
- ☐ 35-44
- ☐ 45-54
- ☐ 55-64
- ☐ >65

3 Place of birth
- ☐ UK
- ☐ E Europe
- ☐ S Asia
- ☐ Africa
- ☐ Other

4 Time since arrival UK
- ☐ UK Born
- ☐ <1 year
- ☐ 1-5 years
- ☐ >5 years

B) Health

5 Do you have any of the following health problems? (mark any)
- ☐ Asthma
- ☐ I use an inhaler
- ☐ Chronic breathing problems
- ☐ Heart problems
- ☐ Stroke
- ☐ Diabetes
- ☐ Hepatitis
- ☐ HIV
- ☐ I was treated previously for TB
- ☐ Epilepsy
- ☐ Mobility problems

6 Do you have any of these symptoms at the moment? (mark any)
- ☐ Weight loss for no reason
- ☐ Coughing for more than 3 weeks
- ☐ Fever or night sweats
- ☐ Coughing up blood
- ☐ Shortness of breath
- ☐ None of these symptoms

7 Are you registered with a GP? (mark one)
- ☐ Yes - Local (easy to travel to)
- ☐ Yes - but not local (hard to travel to)
- ☐ No

8 When did you last see a GP? (mark one)
- ☐ <1 year
- ☐ <2 years
- ☐ Should have done but missed appointment
- ☐ Don't know/remember

9 Have you attended A&E in the last year? (mark one)
☐ No ☐ Once ☐ Twice ☐ 3 times ☐ >3 times ☐ Don't know/remember

10 Have you been admitted to hospital in the last year? (mark one)
☐ No ☐ Once ☐ Twice ☐ 3 times ☐ >3 times ☐ Don't know/remember

11 Have you missed any hospital appointments in the last year? (mark one)
☐ No ☐ Once ☐ Twice ☐ 3 times ☐ >3 times ☐ Don't know/remember

C) Blood Born Viruses

12 Have you been vaccinated against Hepatitis B? (mark one)
☐ Never ☐ Once ☐ Twice ☐ 3 times ☐ >3 times ☐ Don't know/remember

13 Have you been tested for HIV? (mark one)
☐ Yes ☐ No ☐ Don't know/remember

14 Have you been tested for Hepatitis C? (mark one)
☐ Yes ☐ No ☐ Don't know/remember

D) Flu Vaccination

15 Did you know there is a seasonal vaccine to prevent influenza?

- [ ] Yes
- [ ] No
- [ ] Don't know/remember

16 If YES where did you find out about flu vaccine?

- [ ] Hostel
- [ ] GP
- [ ] Hospital
- [ ] Pharmacist
- [ ] Drug service
- [ ] Other

17 Have you ever been vaccinated against flu?

- [ ] Yes - but not last winter
- [ ] Yes - last winter
- [ ] Tried to get vaccinated but was refused
- [ ] No
- [ ] Don't know / remember

18 If YES where did you get flu vaccine? (mark any)

- [ ] Hostel
- [ ] GP
- [ ] Hospital
- [ ] Pharmacist
- [ ] Drug service
- [ ] Other

19 Would you get vaccinated against flu if we could offer it?

- [ ] Yes
- [ ] No

E) Lifestyle

20 Do you get any state benefits? (mark one)

- [ ] Yes
- [ ] No (not applied)
- [ ] No (refused)
- [ ] Not entitled

21 Do you smoke cigarettes? (mark one)

- [ ] Yes
- [ ] Yes (want help to stop)
- [ ] No

22 Do you drink alcohol everyday? [ ] Yes [ ] No

23 Do you drink alcohol soon after waking? [ ] Yes [ ] No

24 Have you ever slept rough? (mark one) [ ] Yes [ ] No

25 Have you ever lived in a hostel? (mark one) [ ] Yes [ ] No

26 Have you ever used a day centre? (mark one) [ ] Yes [ ] No

27 Have you been in prison before? (mark one) [ ] Yes [ ] No

28 Have you used heroin? (mark any)

- [ ] Previously injected
- [ ] Previously smoked
- [ ] Currently inject
- [ ] Currently smoke
- [ ] Never

29 Have you used crack/cocaine? (mark any)

- [ ] Previously injected
- [ ] Previously smoked
- [ ] Currently inject
- [ ] Currently smoke
- [ ] Never

30 Have you been prescribed methadone or subutex? (mark one)

- [ ] Never
- [ ] Previously
- [ ] Currently

**Cepheid Study?** (mark one)	[ ] Yes - MXU number ____________	[ ] No
**Referred from MXU?** (mark one)	[ ] Yes - MXU number ____________	[ ] No
**LTBI Study?** (mark one)	[ ] Yes - Study number ____________	[ ] No

By Placing a tick in one box in each group below, please indicate which statement best describes you own health state today.

MOBILITY

I have no problem in walking about

I have some problems in walking about

I am confined to bed

SELF-CARE

I have no problems with self-care

I have some problems washing or dressing myself

I am unable to wash or dress myself

USUAL ACTIVITIES (e.g. Work, Study, Housework, Family or Leisure activities

I have no problems with performing my usual activities

I have some problems with perfomning my usual activities

I am unable to perform my usual activities

PAIN/DISCOMFORT

I have no pain or discomfort

I have moderate pain or discomfort

I have extreme pain or discomfort

ANXIETY/DEPRESSION

I am not anxious or depressed

I am moderatley anxious or depressed

I am extremely anxious or depressed

To help people say how good or bad a health state is, we have a scale on which the best state you can imagine is 100 and the worst state you can imagine is marked 0.

We would like you to indicate on this scale how good or bad your own health is today, in your opinion. Please do this by marking one of the boxes in the scale below to indicte how good or bad your health is today

Your own health state today

100	☐
95	☐
90	☐
85	☐
80	☐
75	☐
70	☐
65	☐
60	☐
55	☐
50	☐
45	☐
40	☐
35	☐
30	☐
25	☐
20	☐
15	☐
10	☐
5	☐
0	☐

Contact questions

Do you have a mobile phone?	YES / NO
Do you have an email address?	YES / NO
Are you on facebook?	YES / NO
Are you on Twitter?	YES / NO
